# Anticancer Potential of Apigenin and Isovitexin with Focus on Oncogenic Metabolism in Cancer Stem Cells

**DOI:** 10.3390/metabo13030404

**Published:** 2023-03-09

**Authors:** Maryam Ghanbari-Movahed, Sahar Shafiee, Jack T. Burcher, Ricardo Lagoa, Mohammad Hosein Farzaei, Anupam Bishayee

**Affiliations:** 1 Medical Technology Research Center, Health Technology Institute, Kermanshah University of Medical Sciences, Kermanshah 6718874414, Iran; 2 Student Research Committee, Faculty of Pharmacy, Kermanshah University of Medical Sciences, Kermanshah 6718874414, Iran; 3 College of Osteopathic Medicine, Lake Erie College of Osteopathic Medicine, Bradenton, FL 34220, USA; 4 School of Technology and Management, Polytechnic Institute of Leiria, Morro do Lena, Alto do Vieiro, 2411-901 Leiria, Portugal

**Keywords:** apigenin, isovitexin, cancer, stem cells, targeted therapy

## Abstract

It has been demonstrated that cancer stem cells (CSCs) go through metabolic changes that differentiate them from non-CSCs. The altered metabolism of CSCs plays a vital role in tumor initiation, progression, immunosuppression, and resistance to conventional therapy. Therefore, defining the role of CSC metabolism in carcinogenesis has emerged as a main focus in cancer research. Two natural flavonoids, apigenin and isovitexin, have been shown to act synergistically with conventional chemotherapeutic drugs by sensitizing CSCs, ultimately leading to improved therapeutic efficacy. The aim of this study is to present a critical and broad evaluation of the anti-CSC capability of apigenin and isovitexin in different cancers as novel and untapped natural compounds for developing drugs. A thorough review of the included literature supports a strong association between anti-CSC activity and treatment with apigenin or isovitexin. Additionally, it has been shown that apigenin or isovitexin affected CSC metabolism and reduced CSCs through various mechanisms, including the suppression of the Wnt/β-catenin signaling pathway, the inhibition of nuclear factor-κB protein expression, and the downregulation of the cell cycle via upregulation of p21 and cyclin-dependent kinases. The findings of this study demonstrate that apigenin and isovitexin are potent candidates for treating cancer due to their antagonistic effects on CSC metabolism.

## 1. Introduction

Despite vast efforts and noteworthy progress in the treatment of cancer, it remains the primary cause of death worldwide. Normal stem cells are defined via their potential for self-renewal, ability to divide and differentiate, and self-imposed restriction on stem cell numbers. Subtle metabolic derangements can transform a normal stem cell into a CSC, which has no control on the number of stem cells. CSCs constitute a very small number of cells in the tumor and are responsible for tumor growth, progression, and recurrence. Therefore, these subpopulations of cells induce tumor propagation, even after operative therapies, and result in tumor aggression [1]. Thus, CSCs are a logical target and have gained significant attention in the effort to control and treat various cancers.

Cellular metabolism reprogramming has a vital role in tumor initiation, progression, resistance to conventional therapy, and immunosuppression [2]. Altered tumor metabolism has clinical importance as it mediates the resistance of tumors against conventional antitumor agents, and metabolic co-targeting arises as a new, highly promising concept for enhancing the effectiveness of approaches of conventional treatment. The metabolic inhibition of tumor growth via CSC-targeting is of particular interest as these cell populations are accountable for tumor regrowth and maintenance after treatments [3].

Traditional cancer therapies have not been effective against drug-resistant cancers or metastasis. Lately, various dietary compounds derived from natural sources have been indicated to be operative in treating different tumors. Flavonoids are one of the naturally arising polyphenolic compounds which are found to be plentiful in vegetables, tea, fruits, grains, seeds, and several traditional medicinal herbs [4,5]. A number of flavonoids, such as apigenin and its C-glycosyl congener isovitexin, have also been indicated to have a suppressive impact on the survival and self-renewal capability of CSCs of various origins [6,7,8,9,10].

Apigenin (4′,5,7-trihydroxyflavone), a flavone in the group of flavonoids, exists plentifully in onions, nuts, tea, and oranges, as well as other common fruits and vegetables. Lately, apigenin has been scrutinized for its low toxicity and anticancer activities [11]. Apigenin was demonstrated to inhibit different cancers in vitro and in vivo via multiple mechanisms, such as the induction of cell cycle arrest and abrogating cell invasion and migration, triggering the host immune response, and inducing cell apoptosis and autophagy via the activation of the extrinsic and intrinsic pathways [12,13,14,15,16,17,18]. Moreover, in vitro and in vivo studies indicate that apigenin can inhibit tumor-initiating properties [19,20,21].

Isovitexin (apigenin-6-C-d-glucopyranoside) is a naturally derived flavonoid that has been applied in traditional Chinese medicine in order to treat numerous diseases [22,23]. Additionally, isovitexin is a potential inhibitor of multiple tumor cells, such as colon, breast, ovarian, prostate, esophageal, and pancreatic cancer, demonstrating its capability for promoting apoptosis in tumor cells [24,25,26]. Furthermore, it has been indicated that isovitexin can suppress the stemness of cancer cells [6].

Therefore, the purpose of this work was to emphasize CSCs as stewards of tumor progression and resistance to drugs, and how chemoprevention with flavonoids could turn into an operative device for controlling tumor growth. Despite several publications discussing an overview of apigenin and its congener in the context of cancer, these reviews are without specific emphasis on their anti-CSC impacts [11,12,13,14,15,16,17,18,24,25,26]. Henceforth, a critical and extensive review on the anti-CSC capability of apigenin and isovitexin within various cancer types has not been performed before. Accordingly, the present literature evaluation was performed through searching the Scopus, PubMed, ScienceDirect, and Web of Science databases to summarize the results attained from published in vitro and in vivo studies on the impact of apigenin and isovitexin on the eradication of different CSCs.

## 2. Metabolism of CSCs

The initiation of cancer relies on the metabolic reprogramming of oncogenes and tumor suppressors to create a microenvironment capable of fostering unregulated growth. Cancer cells independently change their flux via different metabolic pathways in order to meet the enhanced biosynthetic and bioenergetic demands while mitigating oxidative stress, as is required for cancer cell proliferation and survival. Given that modifications in metabolism precede modifications in stemness, a dysregulation of the CSCs’ metabolic phenotype can be crucial for the CSC state acquirement [27].

Metabolites, when aberrantly gathered, could also enhance tumorigenesis. The improvement in novel technologies over the past decade has not only indicated the tumors’ plasticity and heterogeneity but has also permitted us to discover novel metabolic pathways involved in the support of cancer growth [28]. There is still no consensus on the CSCs’ metabolic characteristics, with different studies demonstrating that they are mostly glycolytic and others instead suggesting mitochondrial metabolism as being their main energy source [29,30]. CSCs also appear to adapt their metabolism to microenvironmental alterations via appropriately shifting the production of energy from one pathway to another, or via obtaining intermediate metabolic phenotypes [31].

**Figure 1:**
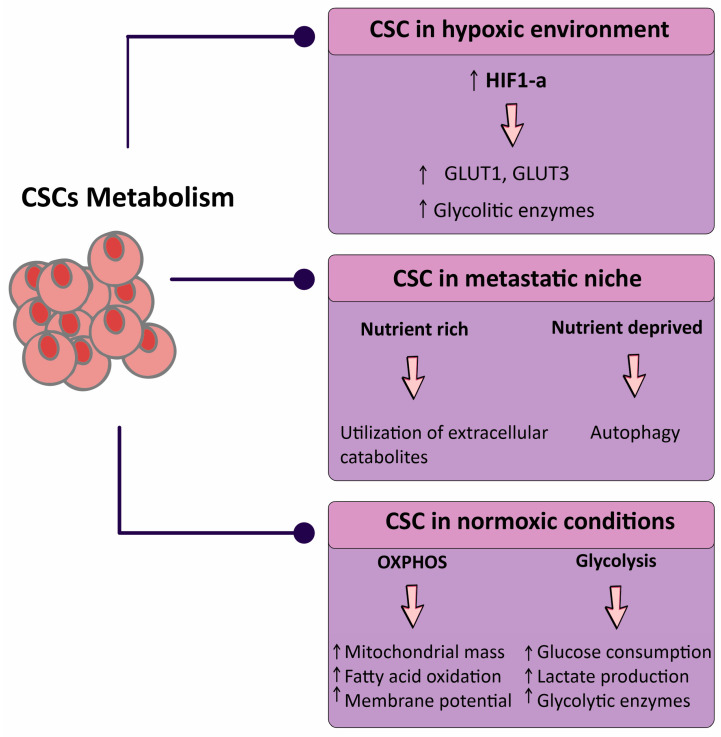
Schematic representation of the metabolic status of cancer stem cells (CSCs) in three broad categories. Abbreviations: CSC, cancer stem cell; GLUT, glucose transporter; HIF, hypoxia-inducible factor; OXPHOS, oxidative phosphorylation. Downward arrow ↓, decrease; Upward arrow ↑, increase.

The impacts of this niche on the metabolism of CSC are similarly beginning to be acknowledged. High catabolism in the microenvironment with hypoxia-inducible factor-1α (HIF-1α), nuclear factor-κB (NF-κB), and transforming growth factor-β (TGF-β) activation coincides with ketogenesis and glycolysis, and enhances CSC features [32,33]. Metabolic stresses also increase major modifications in non-malignant cells inside tumors. T cells are capable to favorably grow into the immunosuppressive regulatory subtype (Tregs) following glucose restriction, which enhances tumor growth [34,35]. Hypoxia modulates the interactions between macrophages and breast CSCs with macrophages polarizing toward an immunosuppressive phenotype with HIF-2α and HIF-1α upregulation (Figure 1) [36,37].

Furthermore, inflammatory interleukins (ILs) generated via the tumor microenvironment (IL-6 and IL-8), along with NF-κB activation, promote a shift towards glycolysis and the activation of phosphatidylinositol-3 kinase (PI3K) and Akt (also known as protein kinase B). These metabolic changes stimulate self-renewal of CSCs, which then might enhance cancer growth and metastasis [38,39,40]. Future research will be required to define the impact of metabolic-altering treatment on tumor cell phenotypes and how this treatment modulates cytokines and signaling pathways to affect CSCs [31].

Therefore, defining the role of CSC metabolism in carcinogenesis has turned into a main emphasis in cancer research, and considerable efforts are directed toward identifying clinical targets.

## 3. Flavonoids and CSCs

Flavonoids are a subclass of polyphenolic compounds which are found plentifully in vegetables, fruits, grains, tea, seeds, and nuts. Many studies have indicated the protective activities of flavonoids in cancer, cardiovascular disease, and age-related illnesses [41]. Accordingly, several flavonoids have demonstrated free radical scavenging, anti-inflammatory, antibacterial, antiviral, hepatoprotective, antiallergic, and antidiabetic activities [42,43]. Several flavonoids have also been demonstrated to possess a suppressive impact on the survival and self-renewal capability of CSCs of different origins [44].

At the metabolic level, flavonoids have been found to modulate signaling pathways that are important for the growth and maintenance of CSCs, such as Notch, Wnt/β-catenin, and Hedgehog pathways. Flavonoids, such as genistein, curcumin, and quercetin, have been indicated to indirectly or directly modulate these signaling pathways and antagonize CSC growth and maintenance [45]. Curcumin (a flavonoid derived from *Curcuma longa*), has exhibited its anticancer impact via suppressing the self-renewal pathways of CSCs through suppressing Notch signaling in esophageal cancer [46]. In an interesting piece of work, genistein reduced mammosphere formation and suppressed stem-like/progenitor cell subpopulations in MDA-MB-231 and MCF-7 breast cancer cells [47]. Another distinguished flavonoid, quercetin, was revealed to induce apoptosis, as well as downregulate stemness proteins and angiogenesis and suppress CSC-derived xenografts in pancreatic cancers [48].

When combined with different chemotherapeutic drugs, flavonoids have revealed synergistic effects for controlling growth, cell survival, maintenance, and stemness of tumor cells and CSCs [49]. The combination of curcumin and the Src inhibitor dasatinib was used toward colon tumor cells enriched with CSCs. The combined treatment showed an improved response in terms of reduced invasion, cell growth, and the colony-forming capability of tumor cells, and also decreased the expression of different CSC markers such as aldehyde dehydrogenases (ALDH), CD166, CD44, and CD133 [50]. Thus, flavonoids are promising molecules against CSCs.

## 4. Apigenin and Isovitexin: Sources, Chemistry, and Pharmacology

**Figure 2:**
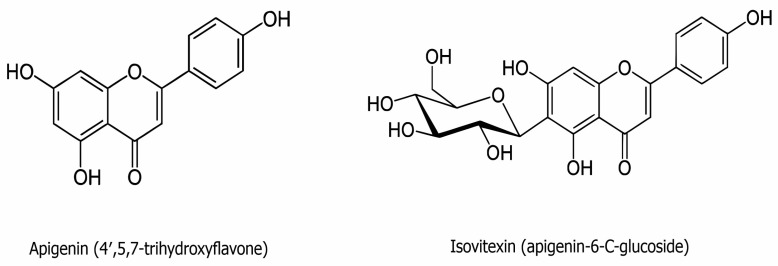
The chemical structure of apigenin (C_15_H_10_O_5_) and isovitexin (C_21_H_20_O_10_).

Flavones are a group of flavonoids characterized by a backbone of 2-phenylchromen-4-one (2-phenyl-1-benzopyran-4-one) (Figure 2). Apigenin is one of the classic natural flavones and is abundant in fruits, vegetables, and beverages, such as parsley, apples, grapes, red wine, and chamomile tea. In its usual sources, apigenin is commonly conjugated to a glycoside [51]. It has low solubility in the aqueous phase but great intestinal permeability, as identified via the single-pass intestinal perfusion method [52].

Apigenin has been applied as a traditional medicine for eras due to its natural antioxidant and anti-inflammatory effects [53,54], ability to lower blood pressure [55], and antiviral and antibacterial activities [56]. Still, apigenin has demonstrated broad antitumor impacts against a multitude of cancers, such as melanoma, osteosarcoma, breast, colorectal, liver, lung, and prostate cancers [57,58,59,60]. This flavone suppresses tumor cell proliferation via inducing cell apoptosis and autophagy and modulation of the cell cycle. Apigenin also reduces tumor cell motility and suppresses tumor cell invasion and migration. In addition, it was demonstrated to regulate the immune response and NF-κB activity, more noticeably in lungs [61]. During tumor inhibition, multiple protein kinases and signaling pathways are modulated via apigenin, such as mitogen-activated protein kinase/extracellular signal-regulated kinase (MAPK/ERK), PI3K/Akt, janus kinase/signal transducers and activators of transcription (JAK/STAT), NF-κB, and Wnt/β-catenin [11].

Isovitexin (apigenin-6-C-glucoside) consists of apigenin with 1,5-anhydro-D-glucitol, a glucoside, substituted at position six (Figure 2). Isovitexin is one of the key active components of the herbs *Passiflora mucronata*, *Cannabis sativa*, *Cucurbitaceae*, *Vigna radiate*, and *Vitex trifolia* L. [22,62]. Some of isovitexin’s pharmacological properties may be attributed to its structure harboring seven hydroxyl groups, and the hydroxyl moiety in the A ring has been suggested to contribute to its proficiency as a free radical scavenger. As with apigenin, isovitexin is a naturally derived flavonoid that has been applied as a traditional Chinese medicine for the treatment of a variety of illnesses [22,23]. The antioxidant and anti-inflammatory activities of isovitexin have been indicated to offer protection against myocardial ischemia–reperfusion injury [63]. Isovitexin is also a potential inhibitor of cancer cells, including colon, breast, prostate, ovarian, esophageal, and pancreatic cancer, exhibiting its ability for apoptosis induction in tumor cells [24,26,64]. Finally, isovitexin has also demonstrated apoptotic and anti-metastatic effects through p53 in human cancer cell lines [26,65].

## 5. Molecular Mechanisms of Apigenin and Isovitexin Involved in Cancer Treatment

**Figure 3:**
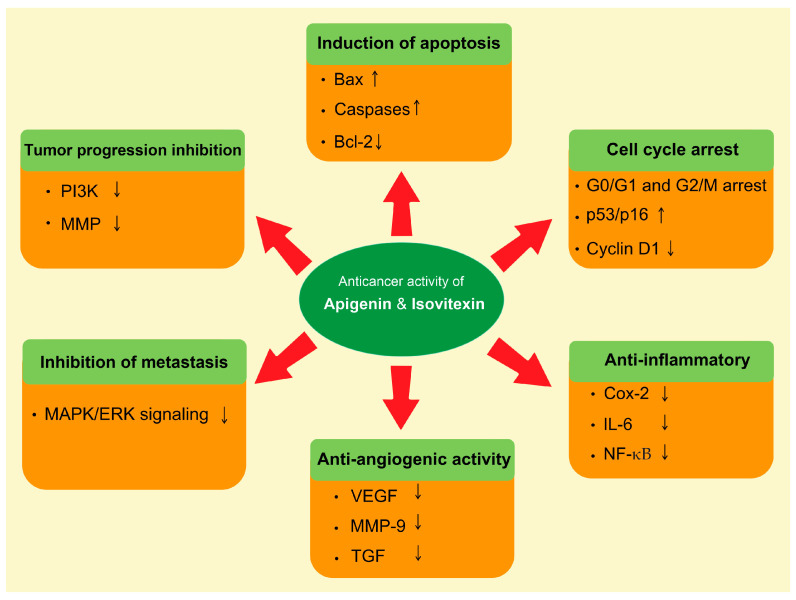
Role of apigenin and isovitexin in cancer management through the modulation of different signaling pathways. The complex cell nature of the tumor is characterized by a number of molecular interactions and mechanisms. Abbreviations: Bax, BCL2-associated x protein; Bcl-2, B-cell lymphoma 2; Cox-2, cyclooxygenase-2; ERK, extracellular signal-regulated kinase; IL-6, interleukin-6; MAPK, mitogen-activated protein kinase; MMP, matrix metallopeptidase; NF-κB, nuclear factor-κB; PI3K, phosphatidylinositol-3 kinase; TGF, transforming growth factor; VEGF, vascular endothelial growth factor. Downward arrow ↓, decrease; Upward arrow ↑, increase.

The natural compound’s role in the prevention of cancer has been rationalized mostly via modulation of the cell signaling pathways [66,67]. Apigenin and isovitexin regulate various molecular pathways, such as apoptosis, autophagy, cell cycle arrest, and angiogenesis, and regulate the expression of various genes (Figure 3).

Apigenin has a noteworthy role in apoptosis induction. It increased apoptosis through the activation of the MAPK signaling pathway, as well as through the reduction in sulfiredoxin expression [68]. Apigenin was demonstrated to increase poly-(ADP-ribose) polymerase (PARP) proteolytic cleavage and to trigger a rapid enhancement of caspase-3 activity. PARP cleavage and DNA fragmentation indicated that apoptosis was enhanced after treatment with apigenin. These effects were related to a modification in the ratio of Bax/Bcl-2 in favor of apoptosis [69]. Apigenin suppressed lung tumor cell proliferation and the activation of vascular endothelial growth factor (VEGF) transcription in a concentration-dependent manner. The mechanism of apigenin-suppressed VEGF transcription was suggested to occur via the reduction in HIF-1α [70].

It has been demonstrated that apigenin enhanced primary effusion lymphoma autophagy and cell death additionally to a substantial reduction in ROS. Moreover, apigenin has been reported to activate p53, which enhances catalase and suppresses STAT3, as evaluated via the silencing of p53 [71].

The administration of apigenin led to G2/M phase cell cycle arrest. The levels of p53 and its protein p21^CIP1/WAF1^ have been found to be enhanced when the cells were treated with apigenin [72]. Additionally, apigenin showed a role in the enhancement of autophagy and apoptosis through the inhibition of the PI3K/Akt/mTOR pathway [73]. Apigenin is also a potential chemopreventive agent that inhibits tumor growth and metastasis via the regulation of the ERK1/2 MAPK and PI3K/Akt signaling pathways [74]. Additionally, through inhibiting the Wnt/β-catenin pathway, apigenin considerably decreased tumor cell proliferation, invasion, migration, and organoid growth [57].

Different authors have indicated that isovitexin induces autophagy and apoptotic cell death of different cancer cells via the upregulation of PARP, Bax, and MAPK, and the downregulation of ERK1/2 and Bcl-2, involving decreased phosphorylation of PI3K, Akt, and mTOR in tumor tissues [26,75]. Experimental findings have demonstrated that isovitexin has anti-inflammatory and antioxidant properties, which influence multiple signaling pathways related to tumor progression and metastatic growth. In addition, isovitexin arrested tumor cell growth at the G2/M cell cycle phase and consequently led to apoptosis induction. This induction of apoptosis appears to be mediated by caspases activation [26].

Moreover, the anti-metastatic effect of isovitexin was demonstrated in CORL-23 and PC12 cells via suppressing HIF-1α and reducing hypoxia-induced genes [76,77].

## 6. Anticancer Activities of Apigenin and Isovitexin against CSCs

Apigenin and isovitexin have been indicated to inhibit different cancers in vitro and in vivo via multiple biological mechanisms, such as enhancing autophagy, cell cycle arrest, and apoptosis, suppressing cell migration and invasion, and modulating signaling pathways such as the MAPK/ERK, PI3K/Akt, JAK/STAT, Wnt/β-catenin, and NF-κB pathways. The anti-CSC activity of apigenin and isovitexin are summarized according to stem cell type in the following sections and can also be found in Table 1 and Table 2.

### 6.1. Brain Cancer

Glioblastoma (GBM) is an extremely malignant human brain tumor with restricted treatment choices. The highly aggressive nature of this disease is due to GBM stem cells (GSCs), a subpopulation in tumors which have self-renewal capacity and resistance to radiotherapy and chemotherapy. Consequently, suppressing GSCs is a logical approach for treating this deadly disease. In one study, the anticancer impacts of apigenin toward the CSC-like phenotypes of human GBM cells U373MG and U87MG were investigated. The results of this study indicated that apigenin considerably inhibited not only the self-renewal capacity of GBM stem-like cells, evidenced by reductions in clonogenicity and cell growth, but also reduced invasiveness. Remarkably, apigenin obstructed the phosphorylation of c-Met and its downstream effectors, Akt, the activator and transducer of transcription 3, and MAPK in the GSCs, thus decreasing the GSC markers’ expression such as NANOG, CD133, and SRY-box transcription factor 2 (SOX2). These findings propose that the GSC suppression impact of apigenin might be triggered via c-Met signaling pathway downregulation [78].

It has been demonstrated that apigenin treatment at the dose of 250–1000 µg/mL inhibited both C6 and U87 cell viability and resulted in major cell cytotoxicity at both 2- and 3-day incubation times. Besides, apigenin considerably eliminated colony formation and cell migration. Overall, the findings demonstrated that apigenin is able to suppress the different stages in glioblastoma carcinogenesis in vitro. So, further investigation of apigenin as an anticancer agent in the clinical treatment of glioblastoma is warranted [79].

### 6.2. Breast Cancer

Compared to other malignancies, breast cancers have increased tumor-initiating capability, more closely recreating the cellular heterogeneity of the original tissue architecture, and generally appear to be more resistant to conventional anticancer therapies. Over the past decade, studies have acknowledged CSCs in solid tumors such as breast cancer that are accountable for tumor initiation, growth, and metastasis [92]. Natural flavonoids, including apigenin, have been shown to increase the therapeutic effectiveness of common chemotherapy agents via the sensitization of CSCs [11].

Treatment with 2–64 μM of apigenin for 48 h significantly inhibited the migration and proliferation of triple-negative breast cancer (TNBC) cells and repressed TNBC cell stemness characteristics both in vivo and in vitro. Treating with apigenin reduced the formation, growth, volumes, and weights of tumors in nude mice bearing MDA-MB-231 xenografts. The investigators attributed these findings to the ability of apigenin to decrease the yes-associated protein/transcriptional coactivator with PDZ-binding motif (YAP/TAZ) activity and the target genes’ expression, such as cysteine-rich angiogenic inducer 61 and connective tissue growth factor, in TNBC cells. They also demonstrated that apigenin disrupted the YAP/TAZ-transcriptional enhanced associate domain protein–protein interaction and reduced the expression of TAZ-sensitized TNBC cells for treatment with apigenin [80].

Sirtuins (SIRTs) overexpression may be another possible therapeutic target for TNBC due to its association with CSC metabolism, resistance to chemotherapy, and metastasis. One study investigated the anti-SIRTs potential of apigenin toward TNBCs. The results of this study indicated that treatment with 12.5–200 µg/µL of apigenin inhibits cellular proliferation via S-phase cell cycle arrest and DNA damage in TNBC cells. Apigenin also suppressed stemness features in TNBCs as indicated in tests of clonogenic and mammosphere formation capability. Mechanistically, researchers concluded that apigenin inhibited SIRT3 and SIRT6 proteins. Collectively, they suggested that apigenin is a promising candidate to be further developed as a sirtuin modulator against TNBCs [81].

In a separate study, treatment with 210 μM for 24–72 h reduced the ALDH-expressing subpopulation of the MCF-7 and JIMT-1 cells through suppression of the tumor necrosis factor-α (TNF-α)-induced NF-κB nuclear translocation [82]. The potential of apigenin to modulate signaling pathways and especially reduce the stemness of breast tumor cells makes it a prime candidate for further examination.

### 6.3. Cervical Cancer

The protein kinase casein kinase 2 (CK2) has been involved in the maintenance of stem cells and its abnormal stimulation has been observed in different types of cancer, such as cervical cancer. Apigenin has also been indicated to possess anti-inflammatory and antioxidant activities [93] and suppress tumor cell metastasis, invasion [94], and MAPK and downstream oncogenes [95]. Comparing it to other flavonoids, apigenin showed high potency to modulate NO metabolism and to inhibit cyclooxygenase-2 (Cox-2) expression in inflammation conditions [94]. In one study, Liu et al. [19] examined the effects of apigenin on the self-renewal capability of sphere-forming cells (SFCs) of the cervical cancer HeLa cells and its basic mechanisms. Based on this study, HeLa-derived SFCs displayed a greater level of CK2α protein in comparison to the parental cells. The results indicated that treatment with apigenin led to dose-dependent inhibition of the self-renewal capability of HeLa-derived SFCs, as well as a reduction in the expression of CK2α. Additionally, forced CK2α overexpression led to a reduction in the suppression of CK2α and the self-renewal capability triggered via apigenin in HeLa-derived SFCs. These findings recommended that apigenin suppresses the self-renewal capability of HeLa-derived SFCs via downregulating the expression of CK2α.

### 6.4. Colon Cancer

Colorectal cancer (CRC) is a main cause of morbidity and mortality all over the world. CSCs have a critical role in the recurrence and metastasis of CRC [96]. Apigenin has been revealed to independently exert tumor-suppressive and anti-inflammatory effects in the context of colorectal cancer. In one study, Zhang et al. [83] explored the impact of chrysin, another flavone, and apigenin on CRC and its associated mechanism. Colorectal cancer cells HCT-116 and SW480 were treated with apigenin and/or chrysin at different doses of 5–100 μM. The results of this study indicated that combined treatment with apigenin and chrysin considerably decreased the numbers of cell clone, invasion, and migration ability, while also increasing cell apoptosis in both cell lines. The combined impact was higher than apigenin or chrysin alone. Researchers found p-AKT and p-p38 to be considerably downregulated by apigenin and chrysin treatment. Apigenin plus chrysin combination therapy demonstrated synergetic effects in suppressing the metastasis and growth of CRC cells via inhibiting the activity of the p38-MAPK/AKT pathway.

### 6.5. Head and Neck Squamous Cell Carcinoma

Head and neck squamous cell carcinoma (HNSCC) is the sixth most prevalent cancer all over the world. CSCs contribute to the recurrence of tumors, and a hypoxic environment is vital for maintaining CSCs. In a study by Ketkaew et al. [84], the impacts of apigenin on the CSC markers of expression in HNSCC cells under hypoxic conditions were investigated. Apigenin considerably reduced HN-30 cell proliferation in time- and dose-dependent ways. Additionally, 40 μM apigenin considerably downregulated the mRNA expression of NANOG, CD105, and CD44. Furthermore, the hypoxia-induced enhancement in CD105^+^, STRO-1^+^, and CD44^+^ cells was significantly eliminated via apigenin. Apigenin inhibits the expression of CSC markers and the cell surface markers expressing cells under hypoxia.

### 6.6. Leukemia

The CD34^+^CD38^−^ leukemia cell population holds leukemia stem cells (LSCs) accountable for therapy failure in acute myeloid leukemia (AML) and, therefore, new therapies are needed for eradicating LSCs without harming healthy hematopoietic stem cells (HSCs). In one study, the consequences of co-treatment with apigenin (LY/Api) and LY294002 (a PI3K/Akt inhibitor) on leukemia cells and primary AML cells were evaluated. Results indicated that LY/Api synergistically enhanced apoptosis in leukemia cells, particularly CD34^+^CD38^−^ leukemia cells. LY/Api-induced apoptosis was accompanied via a disturbance in mitochondrial membrane capability and the activation of caspase cascades. The overexpression of Akt or caspase inhibitor abolished this synergistic stimulation in apoptosis via LY/Api. LY/Api also resulted in the notable downregulation of anti-apoptotic proteins, such as NF-κB, and Bcl-xL in CD34^+^CD38^−^ leukemia cells, but not in healthy HSCs. The suppression of both PI3K/Akt and CK2 pathways might be a potential LSC-targeted therapeutic approach for AML [85].

### 6.7. Liver Cancer

Human hepatic carcinoma is a disease with high incidence tightly associated with substantial mortality owing to recurrence and metastasis after first-line treatment [97]. Hepatic carcinoma stem-like cells (HCSLCs) are responsible for the universal poor prognosis in patients with hepatic carcinoma due to a great capability for tumor initiation, progression, metastasis, and recurrence [98]. Consequently, targeting HCSLCs might be an operative therapeutic approach for treating human hepatic carcinoma [99].

It has been reported that isovitexin suppressed the stemness of human hepatocellular carcinoma (HCC) cells. It has been suggested that isovitexin-mediated miR-34a enhancement triggers apoptosis and eliminates the stemness of the HCC SK-Hep-1 cells. With regard to apoptosis-related proteins, isovitexin increased BCL2-associated x protein (Bax) levels and decreased B-cell lymphoma 2 (Bcl-2) and mantle cell lymphoma-1 protein levels in SK-SC. Additionally, after treatment with isovitexin, ATP-binding cassette subfamily G member 2 (ABCG2), NANOG, and ALDH1 mRNA levels were reduced, while there was a concomitant enhancement in miR-34a levels. These data demonstrated that isovitexin possesses therapeutic potential for HCC treatment [6].

Isovitexin substantially reduced stemness-associated markers and colony and sphere formation rates in cultured HCSLCs via inhibiting manganese superoxide dismutase (MnSOD) and forkhead box M1 (FoxM1) expression. Essentially, isovitexin decreased the expression of the CD133 protein and repressed tumor growth in nude mice bearing HCSLCs. The knockdown of FoxM1 or MnSOD increased the impacts of isovitexin inhibition on stemness and carcinogenicity in HCSLCs. Overexpression of FoxM1 or MnSOD weakened the impacts of isovitexin. Moreover, MnSOD knockdown and isovitexin could suppress FoxM1 reporter activity through reduced binding of E2F transcription factor 1 and/or specificity protein 1 onto FoxM1 promoter. The overexpression of FoxM1 reversed the impacts of isovitexin in combination with the knockdown of MnSOD, without impacting the expression of MnSOD. Furthermore, the knockdown of MnSOD plus thiostrepton, a FoxM1 specific inhibitor, collaborated with isovitexin to inhibit tumor growth and reduce FoxM1 and MnSOD in nude mice bearing HCSLCs from the MHCC97H cell line [86]. These results demonstrate that isovitexin inhibits stemness and carcinogenicity in HCSLCs via downregulating FoxM1 through MnSOD suppression.

### 6.8. Lung Cancer

Lung cancer is one of the commonest neoplasms in both developing and developed countries [100], with non-small cell lung cancer (NSCLC) accounting for almost 75–85% of all cases [101]. In spite of sustained improvements in first-line treatment and diagnosis, prognosis remains very poor. At present, treatment modalities are still far from yielding ideal results and new therapies are required to decrease the impact of the growing incidence of pulmonary neoplasms. Apigenin shows modest anticancer activities in vitro and in vivo against lung cancers. In one study, interactions between apigenin and the tumor necrosis factor-related apoptosis-inducing ligand (TRAIL) in NSCLC cells were evaluated. The investigators observed a synergistic impact among apigenin and TRAIL, resulting in the increased apoptosis of NSCLC cells. Notably, lung cancer cell lines H1299 and A549 were resistant to treatment with TRAIL alone. The presence of apigenin sensitized NSCLC cells to TRAIL-induced apoptosis via enhancing death receptor 4 and 5 levels in a p53-dependent way. Additionally, treatment with apigenin led to a reduction in colony formation ability. Consistent with these findings, the anti-apoptotic proteins Bcl-2 and Bcl-xl were reduced, while the pro-apoptotic proteins Bax and Bad were increased. Meanwhile, apigenin downregulated the activation of Akt, ERK, and NF-κB. Treatment with various small-molecule inhibitors specific to these pathways increased TRAIL-induced cell death, reflecting the impact of apigenin. Additionally, using a mouse xenograft model, researchers displayed that combination treatment with apigenin and TRAIL completely suppressed tumor growth compared to monotreatment. These findings demonstrate a new approach for enhancing TRAIL-mediated anticancer activity in NSCLC cells via apigenin through the suppression of the Akt, NF-κB, and ERK pathways [87].

Li et al. [88] tried to clarify whether apigenin is able to enhance the antitumor effectiveness of cisplatin (CDDP) in lung cancer via CSCs. Lung CSCs were recognized as CD133^+^ cancer cells in the NSCLC cell line H1299, A549 cells, and CDDP-resistant NSCLC A549R cells. The apigenin cytotoxic impact was evaluated in CDDP-treated H1299, A549, and A549R cells. Apigenin suppressed CD133^+^ cells and increased the anticancer impact of CDDP in H1299, A549, and A549R cell lines. The synergistic anticancer impact of apigenin and CDDP was inhibited via adding the p53 inhibitor Pifithrin-α and small interfering RNA (siRNA) targeting the *p53* gene in the A549R cell line. Furthermore, apigenin eliminated CDDP-induced CSC via p53. The results demonstrated that apigenin may eliminate CSCs and increase the antitumor impacts of CDDP in NSCLC through p53.

It was found that isovitexin blocked MnSOD/calcium/calmodulin-dependent protein kinase II (CaMKII)/the adenosine monophosphate-activated protein kinase (AMPK) signaling axis and inhibited glycolysis in lung cancer stem-like cells (LCSLCs), leading to stemness suppression in LCSLCs. The MnSOD knockdown considerably increased the isovitexin-associated suppression of CaMKII/AMPK signaling, stemness, and glycolysis in LCSLCs. Nevertheless, the MnSOD overexpression can weaken the isovitexin inhibition on LCSLCs. Essentially, isovitexin eliminated tumor growth in nude mice bearing LCSLCs via downregulating the expression of MnSOD. The isovitexin-associated inhibition of stemness in LCSLCs is partially dependent on the inhibition of the MnSOD/CaMKII/AMPK signaling axis and elimination of glycolysis [8].

### 6.9. Neuroblastoma

Neuroblastoma is the most common extracranial solid tumor in childhood. It frequently rises in the adrenal gland but might also occur in thoracic, abdominal, pelvic, or cervical sympathetic ganglia [102]. For improving the treatments for malignant neuroblastoma patients, novel therapeutic methods must be discovered. In one study, the anti-CSC effects of apigenin against SK-N-BE2 and SK-N-DZ neuroblastoma cells were investigated. The results of this study indicated that treatment with 100 μM of apigenin led to a reduction in cell viability, colony forming ability, and cell survival and enhanced apoptosis by increasing miR-138, caspase-3, and the Bax:Bcl-2 ratio. Moreover, apigenin reduced tumor growth and tumor weight in nude mice bearing SK-N-DZ and SK-N-BE2 tumors [9].

### 6.10. Osteosarcoma

Isovitexin is a flavonoid that displays tumor inhibitory activities on different cancer types. Nevertheless, it is undefined as to whether the mechanism of its action in osteosarcoma (OS) is connected with epigenetic regulation or if it involves microRNAs, DNA methyltransferase 1 (DNMT1), or their targets. It has been demonstrated that isovitexin considerably inhibited survival, enhanced apoptosis, and reduced the levels of CD44, ALDH1, CD133, and ABCG2 mRNA in the spheres derived from MG63 (MG63-SC) and U2OS cells (U2OS-SC). Isovitexin has also exhibited in vivo anticancer activity against osteosarcoma. Isovitexin suppressed tumor growth and decreased the tumor size of U2OS-SC xenografts in nude mice, which was complemented via reduced levels of CD133 protein, raised apoptotic index, reduction in the expression of proliferating cell nuclear antigen (PCNA), decreased DNMT1 expression and activity, enhanced miR-34a, and reduced Bcl-2 levels. Researchers recognized Bcl-2 as being a direct miR-34a practical target. Additionally, isovitexin displayed a synergistic impact with 5-aza-2′-deoxycytidine, the miR-34a mimic, and ABT-263 in order to induce apoptosis, suppress cell survival, downregulate the expression levels of CD44, ABCG2, CD133, and ALDH1 mRNA, and decrease the sphere formation rates of MG63-SC and U2OS-SC cell lines. These results suggest that isovitexin-mediated epigenetic regulation involved the DNMT1/miR-34a/Bcl-2 axis, contributing to the stemness inhibition and apoptosis enhancement in spheres derived from OS cells [89].

### 6.11. Ovarian Cancer

The Hedgehog pathway is involved in CSC growth induction. Its aberrant activation has been identified in different cancer types, such as ovarian cancer. In one study, the SFCs of the human ovarian cancer SKOV3 cells were revealed to have self-renewal capability. SKOV3-derived SFCs had elevated levels of glioma-associated oncogene 1 (Gli1) and CK2α proteins in comparison with those of parental cells. Apigenin considerably suppressed the self-renewal capability and the Gli1 and CK2α protein expression in the SKOV3-derived SFCs in a concentration-dependent way. Furthermore, CK2α siRNA decreased CK2α and Gli1 protein expression and synergistically suppressed the self-renewal capability of the SKOV3-derived SFCs with apigenin. Nevertheless, forced CK2α overexpression led to an enhancement in CK2α and Gli1 expression and decreased the apigenin-suppressed self-renewal impact in the SKOV3-derived SFCs. These findings recommended that apigenin suppressed the SKOV3-derived SFCs’ self-renewal capabilities and was involved in downregulating Gli1 expression via CK2α suppression [90].

### 6.12. Prostate Cancer

Prostate cancer (PC) is the second most common cancer type and the fifth cause of cancer-related mortality amongst males in the United States. The development of chemo-resistance, tumor metastasis, and relapse remain as the main barriers to operative treatment and have all been tied to CSCs [103]. Natural flavonoids such as apigenin have been indicated to have the capability to enhance the therapeutic effectiveness of communal chemotherapy agents via CSC sensitization [104].

The impact of apigenin on apoptosis, cell survival, migration, and stemness in the context of CSCs has been investigated. The results of this study indicate that apigenin, in a dose-dependent way, repressed PC3 cell survival and prostate CSCs, which was complemented with a major enhancement of p27 and p21. Apigenin triggered apoptosis through an extrinsic, caspase-dependent pathway via the upregulation of caspase-8, caspase-3, and TNF-α mRNA expression. Despite this, apigenin was not able to regulate the intrinsic pathway as demonstrated via the cytochrome c (cyt *c*), Bax, and apoptotic protease activating factor-1 (APAF-1) in CSCs. In contrast to CSCs, PC3 cells treated with apigenin experienced upregulated intrinsic apoptosis demonstrated via the stimulation of cyt *c*, caspase-3, and Bax; caspase-8, Bcl-2, and TNF-α levels remained unaffected in PC3 cells. Apigenin strongly suppressed the CSC migration rate in comparison to untreated cells. A major reduction in matrix metallopeptidase (MMP)-2, MMP-9, Slug, and Snail exemplifies the apigenin capability to inhibit invasion. The PI3K, NF-κB p105/p50, and Akt expressions and their phosphorylation were reduced after treatment with apigenin. Furthermore, apigenin considerably decreased the expression of the pluripotency marker Oct3/4 protein which may be connected with the inhibition of the PI3K/Akt/NF-κB pathway [20].

The effects of a combination of cisplatin plus apigenin on CD44^+^ PCa stem cell migration and growth were also evaluated. By adding apigenin (15 μM) to cisplatin (7.5 μM) to cells for 2 days, researchers observed considerably increased apoptotic and cytotoxic impacts compared to cisplatin alone. The investigators attributed these findings to the downregulation of Bcl-2, survivin, and sharpin, as well as to the enhancement of APAF-1, caspase-8, and p53 mRNA expression. The combination therapy reduced p-PI3K and p-Akt, eliminated the expression of the NF-κB protein, and inhibited the cell cycle via p21 upregulation, as well as cyclin-dependent kinase (CDK)-2, CDK-4, and CDK-6. Apigenin considerably enhanced the suppressive impacts of cisplatin on cell migration through a reduction in Snail expression. Briefly, this study indicated the possible adjuvant potential of apigenin to augment the cisplatin impacts via targeting CSCs in prostate cancers [91].

The potential therapeutic application of MK silencing, apigenin treatment, and their combination on human PCa and prostate CSCs (PCSCs) has been evaluated. Both apigenin treatment and MK knockdown led to a loss in PCSCs’ cell viability, and these impacts were considerably enhanced when apigenin was applied to MK-deficient cells. The combined therapy of CD44^+^CD133^+^ PC3 cells with MK siRNA and apigenin was also more operative in triggering apoptotic and non-apoptotic cell death in comparison to individual applications. Treating CD44^+^ LNCaP cells with apigenin considerably reduced cell viability, though the combined treatment did not significantly differ from the individual treatment. Molecular events underlying the suppression of the proliferation, survival, migration, and induction of the cell cycle arrest of CD44^+^CD133^+^ PC3 cells were indicated to be connected with upregulated p27, p21, caspase-3, Bax, Bid, and caspase-8 expression, as well as reduced p-ERK, p-p38, NF-κB, and poly-ADP ribose polymerase [10]. These data indicate that apigenin can be a useful compound for preventing the migration and proliferation of cancer cells, including CSCs.

## 7. Conclusions and Future Perspectives

There is substantial evidence that CSCs trigger tumor perpetuation, even after effective treatments, and support the aggressive features of tumors. Current chemotherapy regimens are not successful in targeting CSCs [44]. Henceforth, there is a necessity for new compounds which are able to target CSCs to attain complete cancer elimination. Moreover, understanding the altered metabolism of tumor cells is of the utmost clinical significance as it mediates tumor resistance toward conventional antitumor agents. Accordingly, the characterization of CSC metabolism may provide insights into metabolic co-targeting, an extremely promising concept to improve the effectiveness of conventional treatment methods [3]. Functional foods and their active constituents have been involved in suppressing cancer signaling pathways and targeting CSCs in a large number of preclinical and clinical studies. Flavonoids such as apigenin and isovitexin have antioxidant and anti-inflammatory activities, as well as chemopreventive and antitumor features. The ability of these compounds to target the CSCs of different origins is mediated through alterations in cancer cell metabolism in a multi-modal manner and via the elimination of several stemness-related transcription factors and signaling pathways. The anti-CSC properties of apigenin and isovitexin have been linked to the modulation of different signaling pathways, such as the NF-κB, Wnt/β-catenin PI3K/Akt, and c-Met signaling pathways, and enzyme activities via stem cell stimulation, the induction of apoptosis, and morphological alterations. Therefore, they are effective inhibitors of distant metastasis in cancer, which can be applied as an adjuvant to radiotherapy and chemotherapy for numerous cancers that are resistant to presently available regimens.

The potential limitations of apigenin and isovitexin underscore the necessity for innovative delivery approaches. Consequently, to reach the full competency of apigenin and isovitexin in cancer intervention and prevention, more in-depth mechanistic examinations are needed. Further studies may focus on clarifying molecular targets and signaling pathways that are leading to CSC suppression via these natural compounds. The most important challenge in developing apigenin and isovitexin as novel therapeutic agents is that most of their anticancer evidence is based on in vitro models of cancer, with limited results based upon in vivo or randomized clinical studies. Furthermore, a very small number of studies were conducted which evaluated their anti-CSC properties. Therefore, more research with a special focus on the anti-CSC properties of these compounds is needed.

This is the first study that offers a review of the literature on the mechanisms of action of apigenin and isovitexin against different CSCs, though there are various other studies on the impact of apigenin and isovitexin on cancer cells rather than CSCs. Taken together, based on the results of this review, these active constituents will function as lead compounds for future antitumor drug discovery targeting CSCs as well as other types of cancer cells.

## Figures and Tables

**Table 1:** Potential anticancer stem cell (CSC) impacts and associated mechanisms of action of apigenin and isovitexin based upon in vitro studies.

Cancer Type	Cell Line	Flavone	Concentration and Duration	Anticancer Effects	CSC-related Molecular Mechanisms	References
Brain	U87MG and U373MG	Apigenin	25–50 μM for 2–21 days	↓Self-renewal capacity, ↓cell growth, ↓clonogenicity, ↓invasiveness	↓CD133, ↓NANOG, ↓SOX2, ↓c-Met, ↓Akt	[78]
Brain	C6 and U87	Apigenin	250–1000 for µg/mL 48–72 h	↓Colony formation, ↓cell migration	Not reported	[79]
Breast	MDA-MB-231 and MDA-MB-436	Apigenin	2–64 μM for 48 h	↓Proliferation, ↓migration, ↓stemness features, ↓mammospheres, ↓self-renewal capability	↓YAP/TAZ activity, ↓CTGF, ↓CYR61, ↓YAP/TAZ-TEADs protein–protein interaction	[80]
Breast	TNBCs	Apigenin	12.5–200 µg/µL for 24 h	↓Stemness properties, ↓mammosphere formation, ↓clonogenic potential	↓SIRT3, ↓SIRT6	[81]
Breast	MCF-7 and JIMT-1	Apigenin	210 μM for 24–72 h	↓Stemness, ↓migration	↓TNF-α-induced NF-κB nuclear translocation	[82]
Cervical	HeLa	Apigenin	10–40 μmol/L for 48 h	↓Self-renewal capacity, ↓proliferation, ↓tumorsphere formation	↓CK2α expression	[19]
Colon	CRC	Apigenin	5–100 μM for 2–3 weeks	↓Cell clone numbers, ↓migration, ↓invasion ability, ↑cell apoptosis	↓p-P38, ↓p-Akt	[83]
HNSCC	HN-8, HN-30 and HSC-3	Apigenin	10–100 μM for 6–48 h	↓Cell viability, ↓CSCs	↓*CD44*, ↓ NANOG, ↓*CD105*	[84]
Leukemia	LSCs	Apigenin	2.5–100 µM for 12–72 h	↑Apoptosis, ↑cell death	↓CK2, ↓PI3K/Akt, ↓Bcl-xL, ↓Mcl-1, ↓XIAP, ↓survivin, ↑caspase cascades	[85]
Leukemia	LSCs	Apigenin	0.5–3 µg/µL for 24 h	↑Apoptosis, ↓proliferation, ↓colonogenecity	↓Bcl-2, ↓Ki-67	[7]
Liver	SK-Hep-1	Isovitexin	Not reported	↓Sphere and colony formation, ↓CD44^+^ cell populations	↓ABCG2, ↓ALDH1, ↓NANOG, ↓Bcl-2, ↓Mcl-1, ↑Bax, ↑miR-34a	[6]
Liver	HCSLCs	Isovitexin	5–20 μM for 72 h	↓Sphere and colony formation, ↓stemness-associated markers	↓MnSOD, ↓FoxM1	[86]
Lung	NSCLC, A549 and H1299	Apigenin	20–40 μM for 24 h	↓Colony formation ability, ↑cell growth arrest, ↑apoptosis	↓Bcl-xL, ↓Bcl-2, ↓NF-κB, ↓Akt, ↓ERK, ↑DR4, ↑DR5, ↑Bad, ↑Bax	[87]
Lung	A549 and H1299	Apigenin	5–30 μM for 24–36 h	↓Proportion of CSCs, ↓self-renewal capability, ↑apoptosis	↑p53	[88]
Lung	LCSLCs	Isovitexin	5–160 μg/mL for 24–48 h	↓Stemness features, ↓self-renewal ability, ↓sphere and colony formation	↓MnSOD/CaMKII/AMPK signaling axis, ↓glycolysis	[8]
Neuroblastoma	SK-N-DZ and SK-N-BE2	Apigenin	100 μM for 24 h	↓Cell viability, ↓colony forming ability, ↓cell survival, ↑apoptosis	↑miR-138, ↑Bax:Bcl-2 ratio, ↑caspase-3	[9]
Osteosarcoma	U2OS (U2OS-SC) and MG63	Isovitexin	1–10 μM for 3–14 days	↓Survival, ↓sphere and colony formation, ↑apoptosis	↓CD133, ↓CD44, ↓ABCG2, ↓ALDH1	[89]
Ovarian	SKOV3	Apigenin	10–40 μmol/L for 48 h	↓Self-renewal capacity, ↓proliferation, ↓tumorsphere formation	↓CK2α, ↓Gli1	[90]
Prostate	PCSCs	Apigenin	1.56–100 μM for 48 h	↓Cell survival, ↓migration rate, ↓invasion, ↑apoptosis	↓NF-κB p105/p50, ↓p-PI3K, ↓p-Akt, ↓p-Akt, ↑p21, ↑p27, ↑caspase-8, ↑caspase-3, ↑TNF-α	[20]
Prostate	PCSCs	Apigenin	1.56–100 μM for 24–72 h	↓PCa stem cell growth, ↓migration, ↓cell cycle	↓Bcl-2, ↓sharpin, ↓survivin, ↓p-PI3K, ↓p-Akt, ↓NF-κB, ↑p53, ↑caspase-8, ↑APAF-1, ↑p21, ↑CDK-4, ↑CDK-2, ↑CDK-6	[91]
Prostate	PCSCs	Apigenin	1.56–100 μM for 24–72 h	↓Cell viability, ↓migration, ↑apoptotic and non-apoptotic cell death, ↑cell cycle arrest	↓p-p38, ↓p-ERK, ↓NF-κB, ↓PARP, ↑p21, ↑p27, ↑Bax, ↑Bid, ↑caspase-3, ↑caspase-8	[10]

Abbreviations: AMPK, adenosine monophosphate-activated protein kinase; APAF-1, apoptotic protease activating factor-1; Bax, BCL2-associated x protein; Bcl-2, B-cell lymphoma 2; CaMKII, calcium/calmodulin-dependent protein kinase II; CDDP, cisplatin; CDK, cyclin-dependent kinase; CK, protein kinase casein kinase; CTGF, connective tissue growth factor; CYR61, cysteine-rich angiogenic inducer 61; DNMT1, DNA methyltransferase 1; DR, death receptor; ERK, extracellular signal-regulated kinase pathway; FoxM1, forkhead box M1; Gli1, glioma-associated oncogene 1; HIF-1α, hypoxia-inducible factor 1α; MCL, mantle cell lymphoma; MnSOD, manganese superoxide dismutase; NF-κB, nuclear factor-κB; PARP, poly-ADP ribose polymerase; PI3K, phosphatidylinositol-3 kinase; SIRTs, sirtuins; TGF-β, transforming growth factor-β; TAZ, transcriptional coactivator with a PDZ-binding motif; TNF-α, tumor necrosis factor-α; YAP, yes-associated protein. Downward arrow ↓, decrease; Upward arrow ↑, increase.

**Table 2:** Potential anticancer stem cell (CSC) impacts and associated mechanisms of action of apigenin and isovitexin based upon in vivo studies.

Cancer Type	Tumor Model	Flavone	Dose and Duration	Anticancer Effects	CSC-related Molecular Mechanisms	References
Breast	Nude mice bearing MDA-MB-231 tumor cells	Apigenin	20 µM for 48 h	↓Tumor formation, ↓tumor growth, ↓tumor volumes and weights	↓YAP/TAZ transcriptional activity	[80]
Liver	Nude mice bearing HCSLCs	Isovitexin	10–40 mg/kg for 3 weeks	↓Tumor growth, ↓carcinogenicity	↓CD133, ↓MnSOD, ↓FoxM1	[86]
Lung	Mouse xenograft model	Apigenin	10 μg/injection for 3 weeks	↓Tumor growth	↓NF-κB, Akt, and ERK prosurvival regulators	[87]
Lung	Nude mice bearing LCSLCs	Isovitexin	12.5–50 mg/kg for 2 weeks	↓Tumor growth	↓MnSOD	[8]
Neuroblastoma	SK-N-DZ and SK-N-BE2 tumors in nude mice	Apigenin	10 μg/injection/mouse for 2 weeks	↓Tumor growth, ↓tumor weight, ↑cell death	↑miR-138, ↑Bax:Bcl-2 ratio, ↑caspase-3	[11]
Osteosarcoma	U2OS-SC tumors in nude mice	Isovitexin	10–40 mg/kg for 2 weeks	↓Tumor growth, ↓tumor size	↓CD133, ↓PCNA, ↓Bcl-2, ↓ DNMT1, ↑apoptotic index, ↑miR-34a	[89]

Abbreviations: Bax, BCL2-associated x protein; Bcl-2, B-cell lymphoma 2; DNMT1, DNA methyltransferase 1; ERK, extracellular signal-regulated kinase pathway; FoxM1, forkhead box M1; MnSOD, manganese superoxide dismutase; NF-κB, nuclear factor-κB; PCNA, proliferating cell nuclear antigen; YAP, yes-associated protein; TAZ, transcriptional coactivator with a PDZ-binding motif. Downward arrow ↓, decrease; Upward arrow ↑, increase.

## Abbreviations

ABCG2	ATP-binding cassette subfamily G member 2
AML	acute myeloid leukemia
AMPK	adenosine monophosphate-activated protein kinase
APAF-1	apoptotic protease activating factor-1
Bax	BCL2-associated x protein
Bcl-2	B-cell lymphoma 2
CaMKII	calcium/calmodulin-dependent protein kinase II
CDDP	cisplatin
CDK	cyclin-dependent kinase
CK	protein kinase casein kinase
CRC	colorectal cancer
CSCs	cancer stem cells
CTGF	connective tissue growth factor
CYR61	cysteine-rich angiogenic inducer 61
Cyt *c*	cytochrome *c*
DNMT1	DNA methyltransferase 1
DR	death receptor
EGCG	epigallocatechin gallate
ERK	extracellular signal-regulated kinase
FoxM1	forkhead box M1
GBM	glioblastoma
Gli1	glioma-associated oncogene 1
GSCs	GBM stem cells
HCC	human hepatocellular carcinoma
HCSLCs	hepatic carcinoma stem-like cells
HIF	hypoxia-inducible factor
HNSCC	head and neck squamous cell carcinoma
HSC	hematopoietic stem cells
JAK/STAT	janus kinase/signal transducers and activators of transcription
LSCs	leukemia stem cells
LCSLCs	lung cancer stem-like cells
LY/Api	LY294002 (a PI3K/Akt inhibitor) and apigenin co-treatment
MAPKs	mitogen-activated protein kinases
MCL	mantle cell lymphoma
MMP	matrix metallopeptidase
MnSOD	manganese superoxide dismutase
NF-κB	nuclear factor-κB
NSCLC	non-small cell lung cancer
OS	osteosarcoma
PARP	poly-ADP ribose polymerase
PC	prostate cancer
PCSCs	prostate cancer stem cells
PCNA	proliferating cell nuclear antigen
PI3K	phosphatidylinositol-3 kinase
SFCs	sphere-forming cells
siRNA	small interfering RNA
SIRTs	sirtuins
SOX2	SRY-box transcription factor 2
TAZ	transcriptional coactivator with a PDZ-binding motif
TEADs	transcriptional enhanced associate domain
TGF-β	transforming growth factor-β
TRAIL	tumor necrosis factor-related apoptosis-inducing ligand
TNBC	triple-negative breast cancer
TNF-α	tumor necrosis factor-α
YAP	yes-associated protein
